# Current Status and Challenges of Human Induced Pluripotent Stem Cell-Derived Liver Models in Drug Discovery

**DOI:** 10.3390/cells11030442

**Published:** 2022-01-27

**Authors:** Tine Tricot, Catherine M. Verfaillie, Manoj Kumar

**Affiliations:** Stem Cell Institute, KU Leuven, 3000 Leuven, Belgium; tine.tricot@kuleuven.be (T.T.); catherine.verfaillie@kuleuven.be (C.M.V.)

**Keywords:** stem cell-derived hepatocyte-like cells, liver disease modeling

## Abstract

The pharmaceutical industry is in high need of efficient and relevant in vitro liver models, which can be incorporated in their drug discovery pipelines to identify potential drugs and their toxicity profiles. Current liver models often rely on cancer cell lines or primary cells, which both have major limitations. However, the development of human induced pluripotent stem cells (hiPSCs) has created a new opportunity for liver disease modeling, drug discovery and liver toxicity research. hiPSCs can be differentiated to any cell of interest, which makes them good candidates for disease modeling and drug discovery. Moreover, hiPSCs, unlike primary cells, can be easily genome-edited, allowing the creation of reporter lines or isogenic controls for patient-derived hiPSCs. Unfortunately, even though liver progeny from hiPSCs has characteristics similar to their in vivo counterparts, the differentiation of iPSCs to fully mature progeny remains highly challenging and is a major obstacle for the full exploitation of these models by pharmaceutical industries. In this review, we discuss current liver-cell differentiation protocols and in vitro iPSC-based liver models that could be used for disease modeling and drug discovery. Furthermore, we will discuss the challenges that still need to be overcome to allow for the successful implementation of these models into pharmaceutical drug discovery platforms.

## 1. Introduction

**Figure 1:**
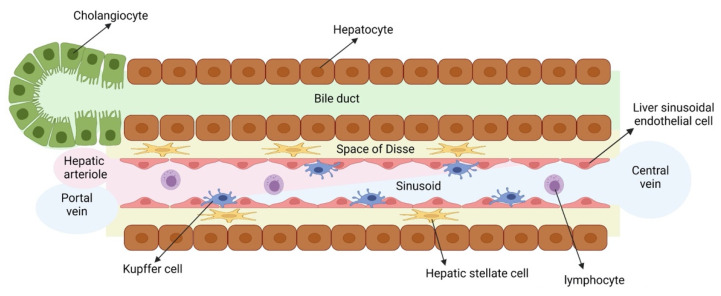
Liver composition. Illustration of the overall cellular composition of a liver lobule.

The liver is the largest organ of the human body and is composed of parenchymal cells, namely hepatocytes and cholangiocytes, and numerous other cells, also called non-parenchymal cells, namely hepatic stellate cells (HSCs), liver sinusoidal endothelial cells (LSECs), Kupffer cells (KCs, the resident liver macrophages) and various immune cells (Figure 1). Hepatocytes in the liver are responsible for multiple vital functions, including the metabolization of drugs, glucose and cholesterol metabolism, glycogen storage and the production of plasma proteins. It is therefore not surprising that liver injury, caused by viral infections, obesity and diabetes (causing metabolic associated fatty liver disease, MAFLD), alcohol abuse, drug intake or congenital diseases can lead to high morbidity and mortality rates in humans [1,2]. Consequently, there is a high need for efficient and relevant in vitro liver models to gain a better understanding of the underlying mechanisms that cause liver injury. These models can also be used for identification of novel therapies to attenuate liver disease as well as the early detection of liver toxicity-inducing drugs and chemicals. To fully recapitulate liver disease in vitro, these models should at least contain hepatocytes. However, if more complex liver diseases (such as fibrosis, steatohepatitis and cholestasis) are to be modeled in vitro, hepatocyte monocultures are insufficient, and more complex in vitro culture systems containing other involved liver cell types (e.g., cholangiocytes, HSCs, LSECs and/or KCs) should be used.

Primary liver cells and cell lines are currently the mainstay for modeling liver diseases and testing drug toxicity. Primary human hepatocytes (PHHs) as well as non-parenchymal cells (NPCs) are the gold standard. However, their limited availability, high batch-to-batch variability, phenotypical instability and tendency to dedifferentiate within 24 to 48 h of 2D conventional cultures (Table 1) present major drawbacks [3,4]. Many efforts have been made to improve the stability of PHHs in cell cultures. This includes the addition of different small molecules to the medium to inhibit pathways involved in de-differentiation [5,6], plating of PHHs between two collagen layers (sandwich culture) [7], micropatterning PHHs with fibroblasts [8] or co-culturing PHHs with primary NPCs, including LSECs, HSCs and/or KCs in 2D or in 3D spheroids [9,10,11,12]. To counteract the activation and acquisition of a myofibroblast phenotype of primary HSCs upon in vitro plating, culturing with a specific growth factor cocktail [13] or co-culturing of HSCs with PHHs or hepatoma cell lines in 3D spheroids [14] has been used. The functionality of primary LSECs that lose their typical fenestrations and eventually undergo apoptosis when cultured alone [15,16], can be prolonged for up to two weeks when co-cultured with PHHs [9]. These techniques have improved the long-term stability of cultured primary liver cells; however, the high batch-to-batch variability and scarce availability remains a significant challenge for the pharmaceutical industry.

Aside from primary cultures, immortalized cell lines have been extensively used for liver disease modeling. As an alternative for PHHs, hepatoma cell lines, such as HepG2, HepaRG and Huh7 cells, have been used for drug toxicity screening or liver disease modeling [17]. However, these are transformed cell lines with, at least for the most commonly used HepG2 cells, low drug metabolizing capacity, rendering them suboptimal for liver disease modeling and drug toxicity testing. As an alternative for LSECs, human umbilical vein endothelial cells (HUVECs) have been used. However, they do not express certain LSEC markers, such as FCGR2B, nor many of the scavenging receptors, and do not display typical fenestrations [18]. In lieu of primary HSCs, hepatic stellate cell lines, such as LX-2, have been used. However, they have an activated phenotype, and are cytogenetically abnormal rendering their application for studying fibrogenic responses questionable [19,20,21].

To overcome the shortcomings of primary liver cells and liver cell lines, the discovery of human pluripotent stem cells (hPSCs), both embryonic stem cells (ESCs) and induced pluripotent stem cells (iPSCs), has created new opportunities in the field of disease modeling and drug discovery [22,23,24,25]. As hPSCs can, in theory, differentiate into any cell of interest, they hold the potential for liver disease modeling and drug discovery. Moreover, hPSCs are highly proliferative, they can be more easily genome-edited than non-dividing primary cells, and can hence be used to create, for instance, stress reporter cell lines [26,27,28] as well as isogenic controls for patient-derived hiPSCs [29,30]. In this review, we will discuss the state of the art of hPSC-derived liver models, their advantages and disadvantages as well as the challenges that remain and how they might be overcome, to allow successful implementation of these models in drug discovery platforms.

## 2. Human Induced Pluripotent Stem Cells as a Model for Liver Diseases and Drug Toxicity

### 2.1. Human Induced Pluripotent Stem Cells

hPSCs are characterized by their ability to self-renew and to generate all the lineage-committed cells of the human body. They were first isolated from the inner cell mass of a blastocyst by Thomson et al. and termed ‘human embryonic stem cells’ (hESCs) [31]. In 2006, Takahashi et al. created so-called ‘induced pluripotent stem cells’ (iPSCs) by reprogramming mouse fibroblasts and a year later, human fibroblasts, via retroviral transduction of the transcription factors *OCT4*, *SOX2*, *KLF4* and *c-MYC* [32,33]. Since the initial description, other reprogramming methods were investigated as retroviral transduction leads to a random integration of the viral vectors into the genome of the host, which may increase the risk of tumor formation and is incompatible with clinical use. These include, non-integrating adenoviral vectors [34] or Sendai vectors [35], virus-free approaches, such as recombinant proteins [36,37], mRNA [38], microRNAs [39] and small molecules [40]. As iPSCs can in theory generate any cell type of the human body, they are believed to be highly suitable for use in disease modeling and drug discovery or toxicity assessment.

### 2.2. Patient-Derived iPSCs and Their Isogenic Controls

Multiple liver diseases have been modeled in vitro by reprogramming patient-derived somatic cells towards iPSCs, followed by lineage differentiation towards the appropriate liver progeny [41]. Patient-derived iPSCs have been generated from patients suffering from, e.g., Alpha-1-antitrypsin deficiency [42,43], Crigler Najjar syndrome [44], Familial hypercholesterolemia [45], autosomal recessive hypercholesterolemia [24], Niemann–Pick disease type C [29,46]. When differentiated to their hepatocyte progeny, they must be used for disease modeling and identification of candidate therapeutic drugs. However, in such studies, appropriate controls for the patient-derived iPSCs are crucial. The generation of genome editing tools, initially zinc-finger nucleases (ZFN) and transcription activator-like effector nucleases (TALENS), and more recently clustered regularly interspaced short palindromic repeats (CRISPR)/CRISPR-associated system (Cas9) has allowed to create genetically corrected iPSCs to study monogenic diseases (reviewed in [47]). These isogenic control iPSCs share a single genetic background with the patient-derived iPSCs, which offers the opportunity to eliminate the biological noise, resulting from genetic variability between different hiPSC lines, and thus, are the gold standard control for their diseased counterparts.

### 2.3. iPSC Reporter Lines

As hPSCs are highly proliferative, editing their genome is more readily feasible compared to primary human liver cells that have limited expansion capacity. Therefore, they are amenable to the creation of fluorescent stress pathway reporters [26,27,28,48,49,50,51]. These reporters can be generated by introducing an exogenous promoter and reporter gene in ‘safe harbor’ loci, such as the adeno-associated virus integration site 1 (*AAVS1*) locus [28,52], the citrate lyase beta-like gene locus (*CLYBL*) [53] or the *ROSA26* locus [54] in iPSCs. However, it must be noted that even though the transgene is inserted in a ‘safe harbor’ locus, upon differentiation, silencing may occur in a lineage-specific manner [28]. The use of insulator elements may overcome the problem of transgene silencing during lineage specification [28,55]. As an alternative, knock-in reporter PSC lines can be created whereby a fluorescent reporter gene is inserted at the beginning (after the start codon) or at the end (before the stop codon) of and in frame with the coding sequence of the gene of interest via homologous recombination. Between the coding sequences of the gene of interest and the reporter gene, a linker may be added, which will lead to the formation of a fusion protein of the gene of interest and the fluorescent reporter. This allows monitoring of not only the presence of a specific protein but also the sub-cellular localization of the protein of interest. As an alternative for the linker sequence, an internal ribosomal entry site (IRES) or a 2A peptide sequence can be used, which will lead to the translation of two separate proteins [48,49,51,56]. These fluorescent reporter lines allow for the monitoring of cellular pathways, studying the mode of action (MOA) of drug toxicity or to investigate in vivo transplanted PSC-derived progeny [26]. Furthermore, the fluorescent reporter lines can also be used as an easy readout for chemical and genetic screenings as well as for drug discovery and toxicity assays [27]. Finally, when creating complex liver models with multiple cell types, reporter lines allow easy discrimination of the different cell types.

## 3. Differentiation of Human Induced Pluripotent Stem Cells towards the Different Liver Cell Types

### 3.1. Hepatocytes

**Figure 2:**
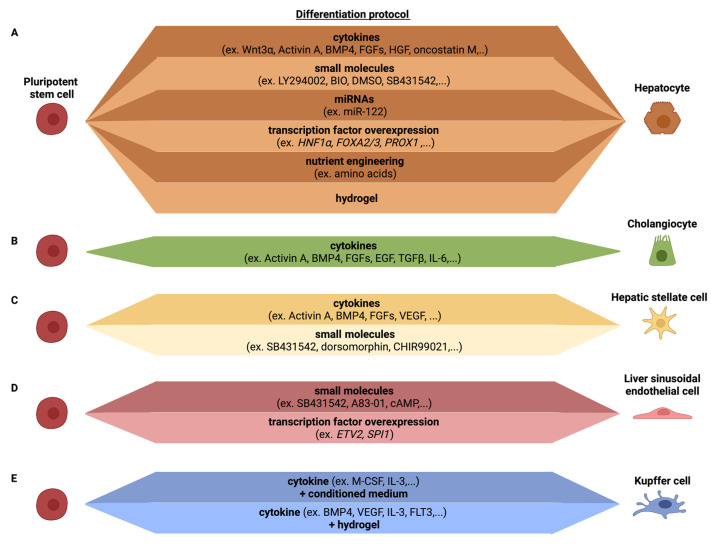
Different protocols to differentiate hPSCs towards (**A**) hepatocytes, (**B**) cholangiocytes, (**C**) hepatic stellate cells, (**D**) liver sinusoidal endothelial cells and (**E**) Kupffer cells.

Hepatocytes are the main parenchymal cell type of the liver, constituting over two-thirds of the liver mass. Hepatocytes are involved in a number of vital functions, including protein synthesis, detoxification and metabolism of lipids and carbohydrates. As they are essential for the liver function, most efforts have been aimed at developing efficient differentiation protocols for hepatocytes from PSCs (Figure 2A).

Current in vitro hepatic differentiation protocols attempt to mirror the in vivo liver developmental processes using cocktails of growth factor to recreate three essential steps: definitive endoderm differentiation, hepatoblast differentiation and hepatocyte maturation. The majority of differentiation protocols use combinations of activin A, Wnt-3A, bone morphogenetic protein (BMP)-4, fibroblast growth factors (FGFs), hepatic growth factor (HGF) and/or oncostatin M, to recapitulate the sequential differentiation stages [5,57,58,59,60,61,62,63,64]. Additionally, small molecules have been used as more cost-efficient alternatives for cytokines. For instance, with a three-step differentiation protocol using Activin A supplemented with the small molecules LY294002 and bromo-indirubin-3′oxime (BIO), followed by sodium butyrate and dimethyl sulfoxide (DMSO) and a final step of SB431542 with DMSO, Tasnim et al. demonstrated that small molecules could be used to differentiate hPSCs towards HLCs [65]. Furthermore, the addition of DMSO or stauprimide during the differentiation protocol was shown to improve the homogeneity of the HLC differentiation, possibly due to a better initial DE differentiation [66,67,68]. Both cytokine- and small molecule-based protocols produce cells with a number of the characteristics of PHHs, including albumin secretion, glycogen storage, and synthesis of urea [61,69,70]. However, hepatocyte differentiation protocols produce cells that still retain fetal hepatocyte characteristics and some characteristics of in vitro 2D cultured PHHs and are, hence, known as hepatocyte-like cells (HLCs) [71,72,73,74] (Table 2). In particular, the gene expression and enzyme activity of cytochromes P450 (CYPs), required for drug biotransformation, are far lower in hiPSC-derived HLCs than PHHs [74,75,76,77], as well as the expression of phase II enzymes and transporters [72,78,79]. Furthermore, as illustrated by Godoy et al., the bulk transcriptome of PSC-HLCs identifies the presence of cells with features of not only hepatocytes, but also intestine, fibroblast and stem cells [80]. Therefore, differentiation protocols require further optimization such that HLCs can be used as a bona fide alternative to PHHs in the drug discovery pipeline.

To enhance fating of HLCs to the level of PHHs, adapted differentiation protocols have included the addition of small molecules that influence different signaling pathways believed to play a role in PHH development, during the differentiation protocol [65,81,82], the use of medium that has optimized nutrient levels [71,83] or a liver-tailored extracellular matrix (ECM) [84]. Alternatively, cell fating can be enhanced by overexpression of hepatic transcription factors (TFs) [71,85] or inclusion of specific miRNAs during differentiation [86]. These adaptations have improved HLC maturity, which unfortunately still falls short of non-cultured PHHs. As readouts used to assess maturation vary between studies, an easy comparison of the effect of these culture adaptations is not straightforward. It would therefore be of interest for the scientific community to settle on a standard set of readouts, used by all labs, to address which of the many possible adaptations induces the most significant HLC maturation to a degree that the improved HLCs are acceptable to regulators/regulatory bodies. Finally, considerable differences in differentiation efficiencies have been observed among different iPSC lines [87], which also needs to be addressed if one wants to efficiently model hepatocyte disorders using patient-derived and isogenic iPSC-HLCs.

### 3.2. Cholangiocytes

Cholangiocytes are a heterogeneous population of epithelial cells that line the network of bile ducts known as the biliary tree [88]. The main function of biliary epithelial cells consists in modifying the primary bile produced by hepatocytes and transport the bile to the gall bladder and gut. Modification of bile occurs via a combination of ion, solute and water transport across the cholangiocyte basolateral and apical membranes.

Similar to hepatocytes, cholangiocytes are derived from bipotential endodermal hepatoblasts during development. When iPSC are fated to the hepatocyte lineage, co-development occurs of CK7^+^ and CK19^+^ cells, which are well known cholangiocyte markers [89,90]. The first defined protocol for cholangiocyte differentiation from hPSCs was published by Dianat et al. [91]. Since then, a number of protocols have been developed to specify bipotent hepatoblasts to cholangiocytes, including the use of FGF10 [92], epidermal growth factor (EGF) [91,93], TGF-β and activation of NOTCH signaling [93,94]. The cholangiocyte-like cells generated from hPSCs exhibit gamma glutamyl transferase and alkaline phosphatase activity and stain positive for acetylated alpha-tubulin, present in primary cilia, characteristic of cholangiocytes. However, iPSC-cholangiocyte-like cells also retain a fetal phenotype as fetal markers, such as *SOX9, HES1* and *JAG1*, remain highly expressed in the hPSC-cholangiocyte-like cells while mature markers *ALP* and *SSTR2* are significantly less expressed than in primary cholangiocytes. Functionally, iPSC-cholangiocyte-like cells shows some similarity to cholangiocyte, including transfer of bile acids, alkaline phosphatase activity, gamma-glutamyl-transpeptidase activity and the cells respond to secretin [91,92,93,94] (Table 2, Figure 2B).

### 3.3. Hepatic Stellate Cells

Hepatic stellate cells (HSCs) are non-parenchymal liver cells responsible for ECM homeostasis and are the main cells involved in the development of liver fibrosis following injury. HSCs are characterized by retinyl-ester containing cytoplasmic lipid droplets [95], and resemble pericytes by surrounding the sinusoids of the liver, thereby regulating blood flow [96]. Following (chronic) liver injury, HSCs activate and transdifferentiate from quiescent, vitamin A-storing cells into proliferative, fibrogenic myofibroblasts, which are primarily responsible for ECM production in fibrotic livers [97,98].

The embryonic origin of HSCs still remains elusive and is a subject of debate [99], even if their mesodermal origin has gained wide acceptance [100]. In contrast to PSC–HLC differentiation protocols, only a limited number of PSC–HSC differentiation protocols have been described [101,102,103,104,105] (Table 2, Figure 2C). To generate HSCs, Koui et al. isolated ALCAM^+^ cells from PSC-derived mesodermal progeny (created by the addition of Activin-A, BMP4, bFGF, VEGF, SB431542 and dorsomorphin), followed by inhibition of the Rho signaling pathway to induce an HSC-like cell phenotype. Addition of the Rho pathway inhibitor induced the expression of HSC markers, such as *LRAT*. However, no comparison was made with primary HSC to verify how closely the PSC–HSC-like cells resembled in vivo HSCs, and/or how quiescent the HSC-like cells were. The ability of the cells to develop in activated collagen secreting HSCs was also not addressed [103]. Miyoshi et al. differentiated hPSC towards HSCs using Activin A, BMP4, bFGF and CHIR99021, combined with a doxycycline induced overexpression of *LHX2.* The overexpression of *LHX2* resulted in an increased expression of genes related to HSC quiescence (e.g., *LRAT* and *NGFR*). However, genes related to HSC activation (e.g., *ACTA2* and *COL1A1*) were not decreased. The resulting HSC-like cells were able to store vitamin A; however, no further functional tests were performed [104]. In contrast, Ouchi et al. generated hepatic liver organoids (HLOs), containing cells with an HSC-like phenotype, by differentiating hPSCs towards foregut spheroids, that were subsequently embedded in Matrigel and treated with retinoic acid. The resulting HLOs contained a mixture of cells of which some had HSC-like features. These HSC-like cells were enriched for HSC markers *VIM*, *CYGB*, *DES* and *COL1A1,* and showed vitamin A storage abilities. However, no comparison with primary HSCs or the quiescent or activated state of the cells was performed [105]. Our lab used a growth factor cocktail approach, including FGF1, FGF3, palmitic acid and retinol, to fate BMP-4-derived mesodermal progeny towards HSC-like cells, without intermediate FACS sorting [101,102]. This generated HSC-like cells with phenotypic and functional characteristics of HSCs. The protocol yields ± 78% PDGFRβ^+^ cells of which 80% store vitamin A, a typical feature of quiescent HSCs. When compared with freshly isolated quiescent human HSCs, PSC–HSC-like cells expressed significantly lower levels of *LRAT*. Consistently, the transcriptome of PSC–HSC-like cells was intermediate between that of quiescent and activated primary HSCs. Nevertheless, following exposure to the profibrogenic transforming growth factor (TGF)-beta-1, PSC–HSC-like cells secreted collagens and upregulated α-SMA expression, and lost expression of *LRAT* and vitamin-A droplets [84,101,102].

### 3.4. Liver Sinusoidal Endothelial Cells

Liver sinusoidal endothelial cells (LSECs) are highly specialized endothelial cells that form a permeable barrier between blood and the liver parenchyma. Unlike other endothelial cells, LSECs lack a basement membrane and are fenestrated, which allows macromolecule transport from the blood to hepatocytes. In addition, they display very high endocytosis capacity important for nutrient exchange and clearance of waste products. Under physiological conditions, LSECs regulate and maintain hepatic vascular tone. They have important roles in innate and adaptive immunity to maintain immune tolerance, express toll-like receptors and inflammasome components, are involved in trafficking of leukocytes, and clear microbial antigens and viruses (reviewed in [106]). LSECs also play a major role in liver disease and regeneration [107,108]. LSECs contribute to persistence of chronic viral infections. They drive initiation and exacerbation of fibrosis, wherein they undergo capillarization, characterized by loss of fenestrae, acquisition of a basement membrane and endothelial-to-mesenchymal transition. LSEC also secrete angiocrine factors that balance regeneration vs. fibrosis.

Despite the important role of LSECs in liver homeostasis and disease, very few protocols have aimed to create cells with LSEC features from PSCs [103,109,110] (Table 2, Figure 2D), even if multiple methods have been described to create PSC-derived endothelial cells [111,112]. Using mouse PSC, Arai et al. already demonstrated in 2011 that cells with LSEC features could be generated from embryoid bodies by the modulation of andrenomedullin-RAMP2 signaling. They showed that co-administration of adrenomedullin and SB431542, a TGFβ receptor type I inhibitor, resulted in an enhanced differentiation of LYVE1/STAB2^+^ EC population. These LSEC-like cells showed a robust endocytosis potential, expressed increased levels of *F8*, *Fcgr2b* and *Mrc1* and possessed fenestrae-like structures [113]. Koui et al. were the first to describe a protocol to create cells with LSEC features from human PSCs [103]. They induced mesoderm differentiation from PSCs, as described in the PSC–HSC section above, and isolated FLK1^+^CD31^+^CD34^+^ endothelial cells by FACS. These cells could be expanded ex vivo and then fated to cells with some features of LSECs, by inhibiting TGF-BR1 with A83-01, and culture in hypoxic conditions. This resulted in cells expressing FCGR2B, STAB2, F8 and LYVE1. No functional studies were performed to assess for instance scavenging function, to assess fenestration, and comparisons with primary human LSECs were not performed. Gage et al. used a combination of cAMP, the TGFβ inhibitor SB431542 and hypoxia to generate cells with LSEC features, as shown by expression of *FCGR2B*, *STAB2* and *LYVE1* in in vitro PSC–LESC-like progeny [110]. Although the PSC–LSEC-like cells in vitro did not fully resemble primary LSECs, the LSECs developed fenestrations and scavenger functions following in vivo transplantation. Importantly, single cell RNAseq demonstrated that the grafted hPSC–LSECs showed a similar molecular profile as primary LSECs. Our group used an alternative approach, by identifying TFs that regulate important gene regulatory networks in LSECs, but not in other endothelial cells or other liver cells, using a combination of bulk and single cell transcriptome studies. Among those TFs, we identified the *SPI1* (encoding for the PU.1 protein), which regulates expression of many LSEC-specific immune functions. To create LSECs, we therefore not only overexpressed the *ETV2* TF, which induces an endothelial cell fate from PSCs [114], but also *SPI1*. This resulted in the creation of endothelial cells that expressed a number of typical LSEC scavenging receptors, such as *FCGR2B* and *MRC1*. More importantly, these scavenging receptors were functional as they supported uptake of FSA-FITC as well as labelled IgG, and this to the same degree as primary murine LSECs [109]. However, ETV2-SPI1 LSEC-like cells did not display fenestrations, and a full comparison with primary human LSECs was not conducted.

### 3.5. Kupffer Cells

Kupffer cells (KCs) are resident liver macrophages and play a critical role in maintaining liver functions. As innate immune cells, they protect the liver from bacterial infections. Similar to LSECs, they have an elaborate number of scavenging receptors, that are involved in removal of particles, protein complexes, bacteria and cell remnants from the circulation. When they become activated, they can acquire an M1- or M2-like macrophage phenotype, and then secrete cytokines, such as IFNγ, TNFα, IL12, IL23 and IRF5 (M1) or IL4, IL10, IL13, IL33, G-CSF or TGFβ (M2). These factors can then directly activate HSCs, LSECs and hepatocytes [115,116,117]. Due to the unique functions and metabolism of KCs, it has been suggested that they are an attractive target for therapy of liver inflammation and related diseases, including cancer and infectious diseases [115].

In mice, it has been well documented that KCs are derived from yolk sac hematopoietic cells, not from definitive hematopoietic stem cells [118]. However, upon death of these yolk sac KCs, they can be replaced by definitive hematopoietic stem cell-derived monocytes [119]. Whether this is also true for human KCs is less well understood. Multiple studies have been reported wherein hPSCs are fated to the monocyte/macrophage lineage (reviewed in [120,121]). However, only a single study was reported wherein hPSCs were differentiated into cells with KC-like features [122]. hPSC were fated to macrophage precursors using the protocol described by Wilgenburg et al., which is based on embryoid body formation followed by M-CSF and IL-3 addition to generate monocytes, and finally, macrophage precursors [123]. These precursors are subsequently exposed to the hepatocyte culture medium (HCM) and advanced DMEM to induce a KC-like fate. These KC-like cells expressed *CLEC4F* and *SIGLEC1* to similar levels as observed in primary KCs. In addition, PSC–KC-like cells expressed *CD68* and *CD11*, although at levels lower than in primary KCs. LPS and TNFα stimulation induced an inflammatory response similar to that in primary KCs. The differentiation protocol generated in our lab follows a similar approach: macrophage precursors are first produced followed by encapsulation into a defined hepatocyte supportive hydrogel [84]. After 1 month in the hydrogel, the macrophages expressed some KC marker, such as *CD5L*, *MACRO*, *CD14, CD68, CD163, CD11b* and *FCGR2B*. Furthermore, LPS induced a strong inflammatory response. However, direct comparison between these PSC-derived KC-like cells and primary KCs still have to be performed to determine to what extent they capture the KC phenotype (Table 2, Figure 2E).

### 3.6. Liver Resident Lymphoid Cells

The liver also contains a number of liver resident lymphoid cells, including CD8+ T cells, NKT and mucosal associated invariant T cells (so called unconventional T cells), γδT cells, CD8αα intra-epithelial T cells (IELs) and innate lymphoid cells (ILCs), reviewed in [124]. Although protocols for iPSC differentiation into NK cells and different types of T cells have been well described (reviewed in [125,126,127]), few if any studies have attempted to create lymphoid cells with typical liver resident characteristics.

**Table 2:** Assessment of the existing protocols to differentiate hPSCs towards the different cell types of the liver.

Cell Type	Protocol	Characteristics	References
Hepatocytes	Cytokines	Albumin secretion Glycogen accumulation Synthesis of urea Expression immature markers (e.g., *AFP*) Poor CYP450 activity Low expression transporters and Phase II enzymes	[5,57,58,60,61,62,63,64]
	Small molecules		[65,81,82]
	miRNAs		[86]
	Transcription factor overexpression	Albumin secretion Glycogen accumulation Synthesis of urea Expression immature markers (e.g., *AFP*) Improved CYP450 activity	[71,85]
	Nutrient engineering		[71,83]
	Hydrogel		[84]
Cholangiocytes	Cytokines	Presence of primary cilia Gamma glutamyl transferase activity Alkaline phosphatase activity Secretin-induced biliary proliferation Higher expression of immature markers (e.g., *SOX9*) Lower expression of mature markers (e.g., *SSTR2, ALP*)	[91,92,93,94]
HSCs	Cytokines	Expression of *PDGFRβ, ALCAM* Lower expression of *LRAT, ACTA2* Vitamin A storage Collagen secretion upon activation with injury mediators	[84,101,102]
	Small molecules	Increased expression of *LRAT* and *ALCAM* Vitamin A storage	[103]
LSECs	Small molecules	Expression of *FCGR2B*, *STAB2*, *LYVE1* Fenestrations Scavenger functions	[103,110,113]
	Transcription factor overexpression	Tube formation Expression of *FCGR2B, LYVE1* Fenestrations Scavenger functions	[109]
Kupffer cells	Cytokines + conditioned medium	Expression of *CLEC4F*, *SIGLEC1*, *CD11b* and *CD68* LPS induced inflammatory response	[122]
	Cytokines + hydrogel	Expression of markers *CD14, CD68, CD163, CD11*, *CD5L* and *FCGR2B* LPS induced inflammatory response	[84]

## 4. Liver Cell Culture Models: From Simple 2D Cultures to Complex Multicellular 2D Cultures

### 4.1. 2D Culture Systems

The 2D monolayer cell culture is a traditional in vitro model for studying the response of cells to drugs. This model has the advantages of easy and low-cost operation. Multiple studies have been carried out wherein 2D cultured hPSC–HLCs were used for drug toxicity testing and modelling liver disease. Even if hPSC–HLCs have lower drug biotransformation capabilities than short-term cultured PHHs, correct identification of toxic drugs and chemicals appears superior over that of immortalized cell lines, commonly used in pharmaceutical studies [43,71]. Moreover, like for PHHs, hPSC–HLCs can also be used for the identification of drug-mediated steatosis, apoptosis and cholestasis [128]. Due to the relatively easy genome engineering of iPSC, mode of action (MOA) of drugs can be evaluated in real time using HLCs derived from PSCs wherein stress pathway reporter genes have been incorporated, which cannot be achieved using PHHs [51]. 2D hPSC–HLC culture systems exploiting patient-derived iPSC, including genetically corrected isogenic cells, can also be used to model genetic liver diseases and identify new drug candidates for diseases, such as familial hypercholesterolemia, glycogen storage disease, Wilson’s disease, Alpers syndrome, α1AT deficiency, Crigler-Najjar Type 1, defective mitochondrial respiratory chain complex disorder and hereditary tyrosinemia [41,42,44,45,128,129,130,131,132,133,134]. In addition, 2D hPSC–HLC models also allow studying infectious liver diseases caused by Hepatitis B, C and E (reviewed in [135]) or malaria [136]. Finally, such 2D differentiation cultures could be upscaled and automated such that screening of toxicity or efficacy of drugs can be conducted at a medium throughput level (100–3000 compounds) and have been used for drug testing and liver toxicity screenings by different research groups, but are to the best of our knowledge not yet implemented in industrial testing platforms [71,128,137,138,139].

Nevertheless, even though 2D PSC–HLC cultures are an easy and stable model to perform drug screens, they remain immature, express fetal markers, as well as some ‘de-differentiation’ characteristics also observed in long-time plated PHHs [3,4]. HLCs express low levels of a number of phase I and phase II drug metabolization genes and enzyme activity [72,78,79,80]; consequently, for drugs that are metabolized to toxic metabolites by certain *CYP450* genes, toxicity will likely not be detected and this may lead to false-negative or false-positive results. In addition, as not all transmembrane transport proteins responsible for drug uptake are expressed at levels seen in PHHs, this may also affect drug toxicity and efficacy evaluation [78,79]. As with PHH mono-cultures, 2D HLC mono-cultures fail to reflect the complexity of the multicellular liver.

### 4.2. 3D Culture Systems

To maintain PHH maturity and/or enhance and maintain hPSC–HLC maturity, 3D cultures have been developed. Terminology used for such cultures ranges from aggregates or spheroids to organoids. Although in more recent years, these different terms have been associated with specific approaches to create 3D liver (and other tissue) models, many studies use the terms interchangeably. Aggregate or spheroid cultures consist of pre-differentiated lineage specific cells/precursors that are allowed to aggregate and then be maintained in 3D culture. This can be carried out using a single cell type or by incorporating multiple different cell types that can be derived from either one or multiple germ layers. Organoids by contrast, are in general derived from a single cell, whereby spontaneous differentiation and patterning occurs, yielding multicellular cultures that do or do not display a central cavity. Although many organoids contain differentiated progeny from only a single germ layer (e.g., hepatocytes and biliary cells), some reports have suggested that liver organoids may also contain cells of a second mesodermal germ layer, important to recreate the multi-germ layer origin of complex liver tissue.

Spheroid/aggregate cultures or organoid cultures can be generated scaffold-free, or by incorporation in a scaffold. Scaffolds for liver model creation usually are porous, soft hydrogels, that enable free transmission of liquid, gasses and molecules. These scaffolds can be generated from natural polymers (e.g., gelatin, collagen, laminin or mixed ECM components as in Matrigel) or synthetic polymers (such as polyethylene glycol (PEG), polyacrylic and self-assembling peptides), whereby mechanical, physiochemical and biological characteristics can be tailored to support the maintenance of the specific (liver) cells within spheroids and organoids. These hydrogel solutions can also be used as so-called bio-inks when 3D liver models are created using bioprinting.

Using a 3D structure including a number of (non-)parenchymal cell types in the liver allows to better recapitulate the in vivo liver by improving the liver-specific metabolism, as well as partially replicating the organization of the liver, the cell–cell signaling and partial zonation in the liver; these are all factors that can contribute to better prediction of drug toxicity. Unfortunately, the potential for high-throughput screening is more limited due to the labor-intensive setup, which limits the use of these models. Nevertheless, they represent a more functionally relevant model for, perhaps, a secondary toxicological study of candidate compounds.

In the following section, we will discuss simple hPSC–HLC-only 3D cultures and 3D cultures containing multiple different cells derived from hPSCs, created using any of the techniques described above.

#### 4.2.1. 3D Hepatocyte Monocultures

To counteract de-differentiation of PHHs when cultured in 2D in classical culture dishes, PHHs can be cultured as aggregates/spheroids, which then allows preservation of the PHH function for up to 2–3 weeks [140]. These studies were replicated to hPSC-derived HLCs by multiple research groups. For example, Ogawa et al. demonstrated that PSC–HLCs, harvested on day 26 from 2D cultures, could form aggregates. When these aggregates were cultured in the presence of 8 bromo-cAMP, HLCs attained greater maturity, demonstrated by drug metabolization, albumin secretion and transcriptome analysis [141]. Similar results were also described by Takayama et al., who demonstrated that HLC spheroids created from day 11 2D PSC–HLC progeny displayed greater CYP450 activity compared with HLCs maintained in 2D, and that the HLC spheroids could more accurately detect hepatotoxic drugs compared to 3D HepG2 spheroids [142]. Our group recently created HLC aggregate cultures by embedding day 8 PSC-HLCs in a tailor-made PEG based hydrogel, identified by screening >250 combinations of hydrogels with different stiffness, degradation ability and ECM/cell adhesion molecule (CAM) peptide functionalization (which was termed hepatocyte maturation or HepMat hydrogel) [84]. PSC-HLCs in HepMat hydrogels could be maintained for >30–40 days and attained greater CYP3A4 activity compared with 2D cultures. In contrast to the spheroids created without scaffolds, HLCs embedded in HepMat hydrogels created smaller spheroids, and some cells remained as smaller clusters or single cells. As also documented in all HLC spheroid/aggregate culture studies described above, the HepMat-cultured HLCs retained fetal features (including expression of AFP) and CYP450 activity remained lower than that of freshly isolated PHHs. Another example is the study by Ng et al., who developed inverted colloidal crystal PEG scaffolds to embed PSC-derived hepatic progenitors [143]. Improved HLC functionality over 2D cultured HLCs was shown, and following implantation of the spheroid containing hydrogels in murine liver, the porous structure of the crystal PEG-based hydrogel allowed for vascularization.

Thus, although culture of HLCs derived from PSCs in 3D spheroids/aggregates with or without scaffolds improves HLC functionality, the cells remain not completely mature. CYP enzyme activities may be improved compared to 2D cultures but still do not fully reach levels observed in PHHs, which has to be taken into account when performing drug toxicity screens.

#### 4.2.2. 3D Complex Cultures with Multiple Liver Cell Types

An alternative is to create cell models encompassing not only hepatocytes but also additional epithelial cells (e.g., cholangiocytes) as well as non-parenchymal cells, such as endothelial cells, mesenchymal stellate cells and macrophage/Kupffer cells. It has been hypothesized that in such co-cultures the hepatocyte niche might be better recreated, and this should allow for further maturation of hepatocytes, as is seen during liver development and in postnatal liver. Furthermore, the inclusion of multiple liver cell types should enable to liver disease to more closely recapitulate and especially enable improved assessment of drug toxicity as the liver disease or toxicity is seldomly caused by damage to hepatocytes only. Therefore, these complex models are of great clinical interest. Several approaches have been used, including the creation of aggregates/spheroids from pre-differentiated cells and organoid formation from PSCs, either scaffold-free or in scaffolds.

Among the former studies are, for instance, studies whereby aggregates/spheroids are derived from a mixture of PSC-derived progeny and non-PSC-derived cells, for example, Coll et al., cocultured PSC–HSCs with the hepatoma cell line HepaRG [101]. The function of the hepatoma cells was improved, and culture of the iPSC–HSCs in these spheroids induced a more quiescent phenotype compared with 2D cultured PSC–HSCs. Treatment of these spheroids with fibrogenic and hepatotoxic compounds resulted in a fibrogenic response and ECM secretion. Takebe et al. created aggregates of PSC-derived immature HLCs, combined with human umbilical cord endothelial cells (HUVECs) and mesenchymal stromal cells (MSCs), in so-called liver buds [144]. Engraftment of the liver bud in the brain of SCID mouse supported further maturation of the HLCs. Specifically, hepatic cord-like structures and inter-HLC tight junctions were formed, along with functional HLC maturation. In another example, Ramli et al. committed PSCs to endoderm, created aggregates of these foregut cells and induced maturation to a mixture of hepatocytes and cholangiocytes [145]. These spheroids had functional bile canaliculi and troglitazone, known to cause cholestasis, could disrupt bile flow and cause apoptosis. Although culture with excess free fatty acids resulted in structural changes associated with nonalcoholic steatohepatitis (NASH), lack of non-parenchymal cells, such as KCs and HSCs, prevented detection of inflammation and fibrogenesis.

Others created hepatobiliary cultures by directed differentiation from PSC-aggregates, creating so called hepatic organoids. Wu et al. created PSC aggregates that were then subjected to different sequential cocktails of growth factors and signaling molecules to an HLC and cholangiocyte fate (called hepatobiliary organoids, HBOs) [146]. When supplemented with extra cholesterol, these organoids containing central cysts, filled with bile and could be maintained for more than 45 days. HLC maturation was also observed in longer term-maintained HBOs. Guan et al. used a similar approach, but also used Matrigel as a scaffold to create mixed hepatic and biliary organoids [147]. They formed organoids (so-called hepatic organoids, HOs) consisting either of mainly HLCs, mainly cholangiocyte-like cells or a mix of both HLCs and cholangiocyte-like cells. As for the study by Wu et al., HLC functionality improved over time in culture, although the characterization was relatively limited. The authors could successfully use the HO culture system created from a number of patient-derived iPSCs to study mutations in the *JAG1* gene, essential in NOTCH signaling, which clinically causes Tetralogy of Fallot and Alagille Syndrome.

To further increase the complexity of the models, the liver mimics should encompass not only epithelial cells, but also mesodermal non-parenchymal cells, such as HSCs, LSECs, KCs or even lymphoid cells. Several models have been described. In one type of model, PSCs are allowed to differentiate as organoids into both mesodermal and endodermal progeny. Rashidi et al. created aggregates of undifferentiated PSCs and induced hepatic differentiation in 3D [148]. They demonstrated that these 3D PSC-cultures consisted of a central mesodermal core and were surrounded by multiple layers of hepatocytes. These organoids could be maintained for up to 1 year in vitro, and this was associated with progressive loss of AFP secretion but maintained CYP3A activity. They further demonstrated that the organoids could support liver recovery following partial hepatectomy in rodents. A different example is the model created by Ouchi et al., who induced iPSC aggregates to a foregut fate, followed by embedding in Matrigel and further differentiation towards liver cells [105]. Single cell RNAseq (scRNAseq) analysis revealed that the resulting human liver organoids (HLOs) consisted mainly of hepatocytes (~60%); 30% of the cells had a mesenchymal phenotype, while a small number of biliary cells and Kupffer cells were also present. This and a subsequent study also demonstrated that the transcriptome of a fraction of HLO-derived hepatocytes resembles that of primary liver derived hepatocytes [105,149]. The organoids displayed inducible CYP3A4 activity, the mesenchymal compartment displayed vitamin A storage after retinol treatment, and organoids secreted inflammatory cytokines following LPS stimulation. In response to free fatty acid treatment, signs of steatohepatitis were detectable, which could be reversed by treatment with FGF19. They further demonstrated that the HLOs can also be used to study drug toxicity using a medium throughput screen for, e.g., cholestatic and mitochondrial toxic drugs [149]. Furthermore, Guan et al., created a multi-lineage hepatic organoid that expresses the causative mutation for Autosomal Recessive Polycystic Kidney Disease (ARPKD) and showed that organoid develop abnormal bile ducts and fibrosis, a key feature of ARPKD liver pathology. The ARPKD organoids showed increased collagen formation and activation of the PDGFRB pathway. Use of the PDGFR inhibitor, Imatinib and Crenolanib, could successfully decrease collagen accumulation, indicating that this model could be used to test anti-fibrotic drugs [150]. As a final example, our group created similar complex liver spheroids, but by embedding PSC-progeny committed separately to hepatoblasts, endothelial cells, stellate-like cells and macrophages, in the HepMat hydrogel described above [84]. Instead of scRNAseq, multiple iterative labeling for antibody neodeposition (MILAN) was used to define the composition of these co-cultures. As was observed in the multicellular organoid cultures above, among the hepatocyte progeny in the HepMat-based multicellular liver cultures contained AFP^−^/ALB^+^/CYP3A4^+^ mature hepatocytes, AFP^+^/ALB^+^/CYP3A4^−^ intermediate hepatocytes, AFP^+^/KRT19^+^ hepatic progenitors, AFP^−^/KRT19^−^ cells with a cholangiocyte phenotype, some of them forming bile ducts, CD68^+^ macrophages, VIM^+^ mesenchymal cells and CD31^+^ endothelial cells. As for the HLOs, the multicellular HepMat spheroid cultures displayed higher CYP3A4 functionality compared to hydrogel cultures containing only HLCs, and the co-culture system enabled modeling steatohepatitis caused by oleic acid, which could be reversed/blocked by the candidate anti-NASH drugs, obeticholic acid and elafibranor.

Thus, 3D complex cultures with different liver cell types with/without scaffolds has improved HLC functionality substantially compared to 2D cultures or even 3D monocultures, even if not all studies compared 3D HLC monocultures vs. 3D multicellular co-cultures. Although some HLCs in the 3D multicellular co-cultures start to resemble the phenotype of PHHs, a significant fraction of HLCs remain immature. In addition, how closely the NPCs, present in these multicellular co-cultures, resemble primary freshly isolated NPCs is also not yet fully established.

#### 4.2.3. Bioprinting hPSC-Derived Liver Models

As discussed earlier, the hydrogels used to create scaffold-based organoids/spheroids can also be used in combination with bioprinting, which might enable the creation of anatomically more correct liver mimics. Different methods of bioprinting, such as bio-extrusion [151], inkjet bioprinting [152], valve-based bioprinting [153,154] and digital light processing-based bioprinting [155], have already been used to recreate 3D tissue structures from stem cells. Faulkner-Jones et al. was the first to print liver mimics from PSC-derived HLCs [154]. Using microvalve-based printing, they created a 40-layer HLC-containing alginate hydrogel construct and demonstrated that the printed cells express HNF4α and secrete ALB to similar levels of 2D controls. However, no additional tests were performed to demonstrate the maturation degree of the HLCs. In contrast, Goulart et al. printed iPSC-derived HLC spheroids, iPSC-derived endothelial cells and iPSC-derived mesenchymal cells in an alginate/pluronic-based hydrogel at a ratio of 75–20–5%, respectively [156]. They demonstrated that printing HLC spheroids resulted in prolonged metabolic functionality and a reduced EMT transition, compared to printing HLCs in a single cell suspension. However, no comparison with PHHs was performed. Furthermore, Ma et al. created 3D hydrogel-based tricultures consisting of PSC-derived hepatic progenitor cells with endothelial and mesenchymal supporting cells [155]. The three different cell types were printed via the digital light processing based bioprinting, using a gelatin methacrylate bioink, to recreate the hepatic lobular architecture. The gene expression of hepatic markers, such as *HNF4α, TTRI* and *ALB*, were increased compared to 2D and 3D hepatic progenitor mono-cultures, and ALB and urea production were significantly increased.

These studies have shown that bioprinting liver cells is indeed possible; however, more studies need to be performed to address if recreation of the hepatic lobular structure significantly enhances the functionality of HLCs as well as the NPC compartment. Nevertheless, the use of bioprinting may permit higher-throughput fabrication of the 3D in vitro co-culture models, and may offer better reproducibility and precision compared to conventional methods of fabrication of 3D models. This in itself may constitute a major advantage for drug discovery purposes.

### 4.3. Microphysiological Systems

Even though the generation of 3D static cultures has improved the maturation and functionality of the hPSC-derived liver cells, a perfusion system, which facilitates the delivery of nutrients and oxygen to the tissue and re-circulation of endogenous factors, may further aid maturation and specification of the cells in the microtissue and also recreate the typical liver zonation. Microphysiological systems (MPS), also known as ‘organ-on-a-chip’ (OoC), are microfluidic platforms that recreate properties of tissue microenvironments in a dynamic condition. Biomimetic human liver OoCs have advanced from simpler 2D cell models to spheroids or organoids to address the growing need to understand mechanisms of complex diseases, the response to drug exposure and its effect on the tissue [157]. The technology provides spatiotemporal control of the environment and an efficient method for removal of cellular waste products, leading to improved tissue homeostasis. The systems can also allow for the integration of sensors for continuous monitoring. Although OoCs have been developed for culturing primary cells or hepatic cell lines, this technology has also been implemented in the field of PSC-hepatic progeny.

Schepers et al. incorporated day 8 PSC progeny (i.e., hepatoblast) aggregates and inserted these in a microfluidic system. Maintenance of albumin secretion and CYP3A4 activity was demonstrated for up to 3 weeks [158]. Similarly, Wang et al. incorporated PSC-derived embryoid bodies (EBs) in micropillar microfluidic devices and performed in situ differentiation to the hepatic lineage. Resulting organoids contained both HLCs and cholangiocyte-like cells [159]. Perfusion enhanced mature hepatic marker expression (*ALB* and *CYP3A4)* and HLC functionality, including hepatotoxic response to acetaminophen (APAP) in a dose- and time-dependent manner. Similarly, Giobbe et al. differentiated hPSCs directly to hepatocytes and cardiomyocytes in a OoC system and demonstrated a significantly increased functionality as compared with their unperfused control cells [160]. Leclerc et al. demonstrated that perfusion of immature HLCs in microfluidic biochips triggered upregulation of several pathways related to cellular reorganization, stress response and drug metabolism [161].

Hollow fiber bioreactor systems have also been used to differentiate hiPSCs to HLCs [162,163,164]. Miki et al. and Sivertsson et al. differentiated definitive endodermal (DE) derived cells or hepatoblasts towards HLCs in perfused 3D bioreactors [164,165]. This demonstrated that perfusion allowed for improved HLC differentiation compared to conventional 2D cultures, including enhanced CYP expression, albumin and urea synthesis [164,165]. Meier et al. demonstrated that when HLC spheroids were maintained in a hollow fiber perfusion bioreactor, ALB and α1AT secretion improved as well as the functionality of some CYP450 enzymes compared to that of spheroids maintained in static cultures [162]. Furthermore, Freyer et al. reported the formation of bile duct-like structures and improved albumin and urea production in 3D perfusion systems [163]. Recently, Grebenyuk et al. created a microfluidic device that, uniquely enabled by a 3D-printable 2-photon-polymerizable hydrogel formulation, allowed a precise microvessel printing at scales below the diffusion limit of living tissues. This microfluidic system allows long-term perfusion of liver tissue and showed improvement in maturation compared with 2D cultures [166,167]. However, as no comparisons were performed between 3D static and 3D perfused cultures, it remains unclear whether perfusion vs. 3D culture enhanced maturation.

In a further increased complex culture model, Lee-Montiel et al. incorporated PSC hepatic progeny and PSC-cardiac progeny derived from the same iPSC in a functionally coupled OoC system. This enabled the characterization of drug-drug interactions between cisapride and ketoconazole, as the authors could demonstrate that Ketoconazole inhibited the CYP3A4 induced conversion of cisapride to norcisapride, leading to cardiac arrhythmia [168].

The use of perfused microphysiological systems, unlike static cultures, in theory should further improve differentiation and maturation of liver cells form hPSCs. However, currently it is unclear if improved maturation is achieved in perfused vs. non-perfused 3D culture systems, and full maturity has not yet been reached. One of the major benefits of this model is its ability to circumvent fluctuations in parameters, such as pH, oxygen, nutrients and metabolites, which is of interest for drug toxicity testing as these parameters may influence the pharmacokinetics of a tested drug [169]. However, although its complexity could lead to major improvements and advantages for drug discovery, this complexity also makes the model less suitable for high-throughput drug screenings.

## 5. Concluding Remarks

**Figure 3:**
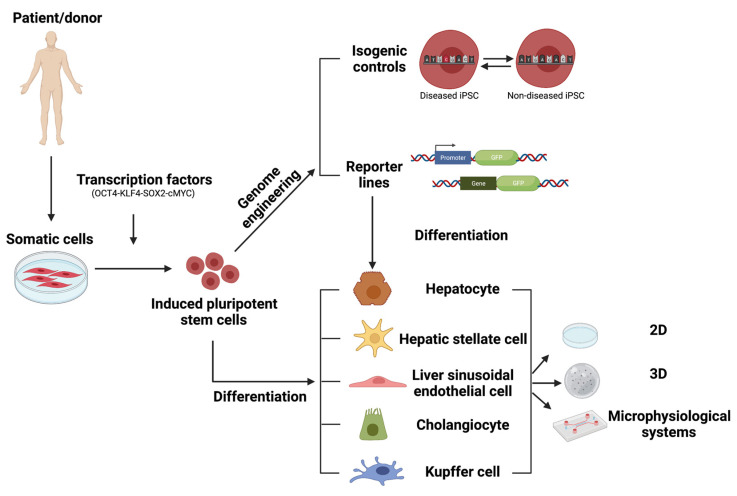
Overview of human induced pluripotent stem cell-derived liver models in drug discovery and liver disease modeling.

hPSC in vitro models have the potential to accelerate drug discovery and development processes (Figure 3). As the highly proliferative hPSCs can be easily genome-edited and, theoretically, hPSCs can differentiate into any cell of interest, they could be used in every stage of the drug discovery pipeline, including in phenotypic screens, target-screens and validation, toxicology evaluation, precision medicine, clinical trial-in-a-dish, and post-clinical studies [170]. In this review, we discussed the state of the art of the hPSC-derived liver models, their advantages and disadvantages as well as the challenges that remain to allow successful implementation of these models into drug discovery platforms.

One of the most prominent and perhaps most challenging limitations of current PSC models is the fact that most PSC-progeny remains immature. Many attempts have been made to achieve more mature liver progeny, e.g., TF overexpression [71,85], medium engineering [71], 3D culture [141,142], co-culture [84,101,103,105] and perfusion [160,162,163,164,165,168]. However, the maturity of the liver progeny requires further thorough investigation with methods, including, for example, single cell/nuclei RNAseq as different cell populations with varying maturity levels might be present in the same culture. A better understanding of this problem is paramount for in vitro modeling and could be transformative for the use of PSC-derived liver models in drug metabolism and toxicity studies, and as disease models. We envision that initial drug discovery screenings could implement the 2D hPSC-derived HLC model as this would be the easiest, most high-throughput and least variable/complex model. Based on candidates from this initial screening, a secondary screen can be performed using 3D complex models that are more accurate and can better recapitulate the in vivo liver cell mixture and function.

Furthermore, although iPSC-derived liver models have been generated, a current critical hurdle is the lack of standardization of these models. It remains somewhat difficult to determine which of the variables used to create any of the liver progeny cultures has superior ability to induce liver progeny maturation, as no side by side comparisons have been carried out, and methods to induce mature functionality are not uniform. Even if many studies now benchmark hPSC-liver progeny against primary cells, the vast inter-donor variability of functionality of the primary cells still impedes the ability of different teams to define which method might induce the greatest maturation across laboratories.

Furthermore, there is a need for further validation of the hPSC-derived liver progeny for potential use in regulatory submissions [171]. The validation process is very lengthy and costly but is still required to confirm how reproducible the models is. Another challenge is to perform multi-centric studies to establish intra- and inter-laboratory reproducibility to ensure that the methodology is fit for purpose, as many of the in vitro models described herein have only been performed in a single lab. Also, there is significant time delay between the development of new in vitro approaches and their acceptance by regulators to replace the existing models. The pharmaceutical industry is reluctant to adopt new methods or approaches unless they are proven equivalent or superior to established models. This represents a significant challenge for rapid implementation of new technologies.

Finally, as most of these models are developed in academic laboratories, the hiPSCs lines are not generated in a GLP environment, which is essential for implementation of the hiPSCs and their liver progeny into drug discovery and development pipelines. Furthermore, the development of a consistent and reliable in vitro translational model requires a set of minimal quality attributes essential for GLP grade iPSCs and their differentiated progeny (reviewed in [172]). In this direction, initiatives are underway by big consortia of both academic and industrial laboratories to address the increasing demand by iPSC researchers for quality-controlled, disease-relevant research grade iPSC lines, data and cell services under one umbrella [173].

To conclude, differentiation of hPSC towards liver progeny is of high interest for liver disease modeling, drug efficacy and toxicity screening. However, a more mature phenotype, standardization and further validation of the liver models will be needed to fully exploit these models in the pharmaceutical industry.

## Figures and Tables

**Table 1:** Characteristics of the different liver cell types.

Cell Type	Markers	Functionality
Hepatocytes	ALB, HNF4α, FXR, PXR Phase -I enzymes, Phase-II enzymes, Transporters	ALB secretion Glycogen accumulation Urea synthesis Drug biotransformation
Cholangiocytes	CK7, CK19, ALP, GGT, SSTR2	Gamma glutamyl transferase activity Alkaline phosphatase activity Secretin-induced biliary proliferation Presence of primary cilia
HSCs	ALCAM, PDGFRβ, ACTA2, LRAT RGS5, IGFBP5, NGFR, CYGB, ADAMST1, GEM	Vitamin A storage Collagen secretion upon activation
LSECs	CD31, FCGR2B, LYVE1, STAB1, STAB2	Tube formation Fenestrations Scavenger functions
Kupffer cells	CD68, SIGLEC1, MARCO, CD5L, CD11, CLEC4F	LPS induced inflammatory response M1 or M2 phenotype Phagocytosis and cytokine release

## Data Availability

Not applicable.
